# Zinc Assisted Thermal Etching for Rich Edge‐Located Fe‐N_4_ Active Sites in Defective Carbon Nanofiber for Activity Enhancement of Oxygen Electroreduction

**DOI:** 10.1002/advs.202407294

**Published:** 2024-08-19

**Authors:** Ruoyu Pang, Hongyin Xia, Xieyiming Dong, Qian Zeng, Jing Li, Erkang Wang

**Affiliations:** ^1^ State Key Laboratory of Electroanalytical Chemistry Changchun Institute of Applied Chemistry Chinese Academy of Sciences Changchun 130022 China; ^2^ School of Applied Chemistry and Engineering University of Science and Technology of China Hefei 230026 China

**Keywords:** defect engineering, electronic structure, oxygen reduction reaction, single‐atom catalysts, zinc–air battery

## Abstract

Single‐atom catalysts (SACs) with edge‐located metal active sites exhibit superior oxygen reduction reaction (ORR) performance due to their narrower energy gap and higher electron density. However, controllably designing such active sites to fully reveal their advantages remains challenging. Herein, rich edge‐located Fe‐N_4_ active sites anchored in hierarchically porous carbon nanofibers (denoted as e_1_‐Fe‐N‐C) are fabricated via an in situ zinc‐assisted thermal etching strategy. The e_1_‐Fe‐N‐C catalyst demonstrates superior alkaline ORR activity compared to counterparts with fewer edge‐located Fe‐N_4_ sites and commercial Pt/C. Density functional theory calculations show that the accumulation of more negative charges near the Fe‐N and the formation of partially reduced Fe state in the edge‐located Fe‐N_4_ sites reduce the energy barrier for the ORR process. Additionally, the unique hierarchically porous structures with mesopores and macropores facilitate full utilization of the active sites and enhance long‐range mass transfer. The zinc–air battery (ZAB) assembled with e_1_‐Fe‐N‐C has a peak power density of 198.9 mW cm^−2^, superior to commercial Pt/C (152.3 mW cm^−2^). The present strategy by facile controlling the amount of the zinc acetate template systematically demonstrates the superiority of edge‐located Fe‐N_4_ sites, providing a new design avenue for rational defect engineering to achieve high‐performance ORR.

## Graphical abstract

By means of the zinc‐assisted thermal etching strategy, the hierarchically porous carbon nanofibers with rich edge‐located Fe‐N_4_ (denoted as e_1_‐Fe‐N‐C) is prepared. The unique electronic properties of edge‐located Fe‐N_4_ with electronic distribution asymmetry cause the polarized surface charges and appropriate adsorption/desorption capacity for oxygen intermediates. Therefore, the edge‐located Fe‐N_4_ site exhibits boosted ORR activity compared with the Fe‐N_4_ site.

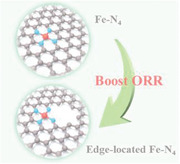

## 1 Introduction

Electrochemical energy conversion and storage devices including fuel cells, secondary batteries, and supercapacitors, have become crucial technologies for addressing the energy crisis due to their environmental friendliness and sustainability.^[^ ^1^ ^]^ Among these, zinc–air batteries (ZABs) have gained significant attention as ideal and reliable energy storage systems because of their ultrahigh theoretical energy density and the abundance of zinc anodes.^[^ ^2^ ^]^ However, the sluggish kinetics of the oxygen reduction reaction (ORR) on the cathode during the discharging process of ZABs have hindered their practical application.^[^ ^3^ ^]^ The benchmark ORR catalysts, such as commercial Pt/C, are not optimal for industrial applications due to their scarcity and inferior stability.^[^ ^4^ ^]^ Consequently, developing high‐efficiency and durable noble‐metal‐free ORR catalysts as alternatives to Pt/C has become a major focus for promoting the application of ZABs.^[^ ^5^ ^]^ Among these alternatives, atomically dispersed single‐atom catalysts (SACs) with well‐defined atomic active centers and maximum atomic utilization have emerged as outstanding and promising catalysts.^[^ ^6^ ^]^ In general, isolated individual metal atoms anchored on a carbon matrix via nitrogen atom bonding, known as metal‐nitrogen‐carbon (M‐N‐C) materials, have captured researchers' attention. Transition metal‐based M‐N‐C materials have demonstrated superior ORR catalytic performance and stability compared to noble Pt/C catalysts, showcasing higher potential for commercial exploitation.^[^ ^7^ ^]^

The *d*‐band configuration of active sites including the *d*‐band center, the *d*‐band electron numbers, and the *d*‐orbital overlap degree is linear to the adsorption strength of the ORR intermediates according to Norskov's theory, which is crucial for regulating the ORR performance of active sites in M‐N‐C catalyst.^[^ ^8^ ^]^ Fe‐N‐C catalysts are the star catalysts toward ORR due to the appropriate *d*‐band configuration of Fe‐N_4_ moieties^[^ ^9^ ^]^. And their activities have also been further improved via the electron redistribution based on the geometrical and electronic modulation.^[^ ^10^ ^]^ Heteroatom doping and the introduction of other metals offer promising options toward more favorable bonding strength between active sites and oxygen‐containing species, but the complexity and variability of doping modes and structures remain a challenge and a topic of debate.^[^ ^11^ ^]^ Besides, defect engineering is also remarkable as an effective means for adjusting the electrical structure and surface morphology, resulting in novel physicochemical properties or powerful synergistic effects that potentially enhance the performance.^[^ ^12^ ^]^ Especially, the construction of defects is more achievable for modulating the electron distribution around single‐atom active sites. Yao's group reported that the role of N‐modified divacancies (ND) configurations in Fe‐N‐C is divided into two models of edge‐ND trapped Fe sites (e‐ND‐Fe) and center‐ND trapped Fe sites (c‐ND‐Fe).^[^ ^13^ ^]^ The e‐ND‐Fe with the higher electron density owing to the electron redistribution and narrower energy gap between the highest occupied molecular orbital (HOMO)‐lowest‐unoccupied molecular orbital (LUMO) than that of c‐ND‐Fe facilitates the electron supply and transfer in catalytic ORR.^[^ ^10^, ^13^, ^14^ ^]^ Up to now, the methods for preparing such edge‐located Fe‐N_4_ sites are primarily focused on removing the metal nanoparticles(NPs) pre‐formed on the carbon matrix by acid leaching.^[^ ^15^ ^]^ These traditional trial‐and‐error methods urgently need more in‐depth consideration and improvement to decrease time‐consuming and laborious efforts^[^ ^16^ ^]^. However, a facile and rational method for controllably preparing edge‐located Fe‐N_4_ sites is still lacking. Beyond the electron redistribution to tune the ORR performance, the accessibility of active sites in catalysts also plays a critical factor for boosting ORR activity. Mesopores and macropores (>2 nm) facilitate local accessibility and long‐range mass transfer, allowing active sites originally buried in the carbon substrate to function effectively.^[^ ^13^, ^17^ ^]^ Therefore, designing more accessible edge‐located Fe‐N_4_ in mesopores and macropores structures holds great potential for achieving a high‐performance ORR response, but little attention has been paid to it.

Herein, a controllable and in situ generation of hierarchically porous carbon nanofibers with rich edge‐located Fe‐N_4_ (denoted as e_1_‐Fe‐N‐C) for boosting electrocatalytic ORR performance was proposed based on the zinc acetate‐assisted thermal etching strategy. The zinc acetate encapsulated in the precursors completely volatilizes during pyrolysis, affecting the ratio of mesopores and micropores, meanwhile, the defects were in situ generated providing the favorable conditions for the acquisition of edge‐located Fe‐N_4_. The optimized e_1_‐Fe‐N‐C material, characterized by abundant edge‐located Fe‐N_4_ sites and a mesoporous/macroporous structure, exhibits superior ORR activity and stability, demonstrating a half‐wave potential (E_1/2_) of 0.876 V versus the reversible hydrogen electrode (RHE) in alkaline media. Moreover, in a zinc–air battery (ZAB) assembly, it achieves a peak power density of 198.9 mW cm^−2^, outperforming commercial Pt/C catalysts (E_1/2_ of 0.841 V and peak power density of 152.3 mW cm^−2^). X‐ray absorption spectroscopy (XAS) analysis confirms the presence of edge‐located Fe‐N_4_ as the active sites responsible for the enhanced ORR activity. Importantly, the impact of varying amounts of zinc acetate on porous structure and defects is meticulously characterized, underscoring the controllability of this thermal etching strategy. This work presents a straightforward and reliable approach for tuning Fe‐N_4_ in a defect‐rich environment within Fe‐N‐C catalysts to boost ORR electrocatalytic activity and performance in ZAB applications.

## 2 Results and Discussion

### 2.1 Synthesis and Characterizations of Catalysts

**Figure 1:**
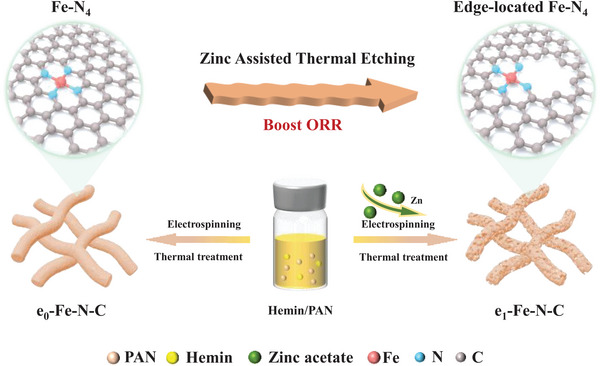
Scheme of the formation of e_0_‐Fe‐N‐C and e_1_‐Fe‐N‐C.

The preparation process of e_x_‐Fe‐N‐C (x represented the amount of precursor zinc acetate dissolved in the electrospinning precursor solution) was illustrated schematically in **Figure** **1**. Initially, polyacrylonitrile (PAN), zinc acetate (Zn(CH_3_COO)_2_·2H_2_O), and hemin were homogeneously dispersed into *N, N*‐dimethylformamide (DMF) to form the electrospinning precursor solution. After electrospinning, the resulting nanofibers were preheated at 500 °C under an ammonia atmosphere for 2 h to enhance nitrogen incorporation, followed immediately by pyrolysis at 1000 °C under an argon atmosphere for 2 h. During this high‐temperature carbonization, the zinc encapsulated in the carbon nanofibers completely volatilized, creating heterogeneous pores. The resulting carbon nanofibers were subsequently treated with 0.5 m H_2_SO_4_ to remove unstable species formed during carbonization, resulting in the formation of e_x_‐Fe‐N‐C. Additionally, metal‐free nitrogen‐doped carbon materials (e_1_‐N‐C) were prepared using the same synthesis method as e_1_‐Fe‐N‐C, but without hemin introduction.

**Figure 2:**
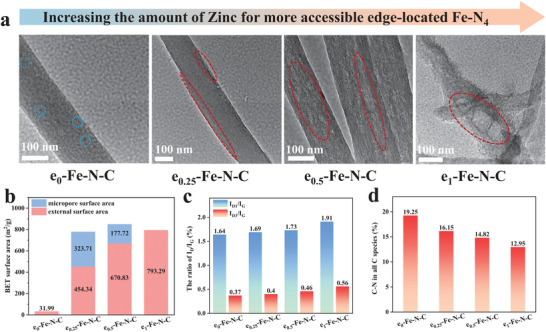
a) HRTEM images of morphological evolution for e_x_‐Fe‐N‐C. b) Summarized specific surface areas of e_x_‐Fe‐N‐C derived by the BET analyses. c) The ratio of I_D_/I_G_ for e_x_‐Fe‐N‐C analyzed by Raman spectra. d) Calculated content ratio of C─N bonds in all C‐species of e_x_‐Fe‐N‐C form High‐resolution C 1s XPS spectra.

As illustrated in Figure S1 (Supporting Information), scanning electron microscope (SEM) images reveal the fibrous morphology of e_1_‐Fe‐N‐C, characterized by a wrinkled surface and an average diameter of ≈200 nm. We observed that the wrinkled surface of e_1_‐Fe‐N‐C, as seen in the transmission electron microscopy (TEM) image in Figure S2a (Supporting Information), is formed by the folding of the carbon matrix. This phenomenon can be attributed to the anisotropic thermal shrinkage of electrospun nanofibers and the outward volatilization of zinc during pyrolysis. To verify the role of encapsulated zinc acetate in controllably adjusting the geometrical structure of the catalysts, additional characterizations were performed on e_x_‐Fe‐N‐C catalysts. As seen in the high‐resolution transmission electron microscopy (HRTEM) images and TEM images of e_x_‐Fe‐N‐C in **Figure** **2**a and S2 (Supporting Information), NPs are not observed with the amount of zinc acetate decreasing from 1 to 0.25 g and sporadic NPs appear only (marked with the blue dashed lines) in the sample of e_0_‐Fe‐N‐C without the introduction of zinc acetate. Obviously, the pore architecture of e_x_‐Fe‐N‐C is significantly affected by the amount of zinc acetate. The pore size of e_x_‐Fe‐N‐C catalysts is positively correlated with the amount of zinc acetate. Specifically, the pore structure is nearly unobservable in the e_0_‐Fe‐N‐C, but it becomes more pronounced and larger as the amount of Zn increases (marked with the red dashed lines), and the pore size is the largest with the highest amount of zinc acetate in e_1_‐Fe‐N‐C. The Brunauer–Emmett–Teller (BET) specific surface area analyses for the e_x_‐Fe‐N‐C samples shown in Figure 2b and Figure S3 (Supporting Information) also confirm this phenomenon. Although the high specific surface areas are almost similar with e_1_‐Fe‐N‐C (793.29 m^2^ g^−1^), e_0.5_‐Fe‐N‐C (848.55 m^2^ g^−1^), and e_0.25_‐Fe‐N‐C (778.26 m^2^ g^−1^) samples, the pores distribution in e_1_‐Fe‐N‐C are all mesoporous/macropores compared to the mesopores/micropores structures of e_0.5_‐Fe‐N‐C and e_0.25_‐Fe‐N‐C, and such enlarged porous structures are thought to be more favorable for the exposure of the active sites and the mass transfer of ORR‐involved reactants. Notably, such porous structure was obtained with zinc acetate as a template rather than hemin, since similar morphology can be observed in the TEM image of e_1_‐N‐C with a high surface area of 863.28 m^2^ g^−1^ (Figures S4, S5, Supporting Information) while e_0_‐Fe‐N‐C has a smooth surface with a low area of only 31.99 m^2^ g^−1^. More impressively, such deliberate design facilitates the generation of defects with edge‐located atomic Fe sites in the catalyst. Raman spectroscopy was then carried out to further investigate the carbon structure changes of e_x_‐Fe‐N‐C samples (Figure 2c; Figure S7, Supporting Information). Raman spectra in Figures S6, S7 (Supporting Information) and for e_1_‐Fe‐N‐C and the contrastive samples show the obvious D band (≈1350 cm^−1^) and G band (≈1592 cm^−1^) belonging to disordered carbon atoms with the vibration mode of A_1g_ symmetry from graphene layer edges and ordered sp^2^‐bonded graphite carbon, respectively.^[^ ^18^ ^]^ In addition, Raman spectra between 1000 and 1600 cm^−1^ can be divided into three kinds of structure configurations for carbon defects, corresponding to D2 band (≈1186 cm^−1^) and D3 band (≈1502 cm^−1^) and the typical D band (denoted as D1), while the D2 band originates from carbon atoms outside of a perfectly planar graphene network and the D3 band is derived from the amorphous carbon.^[^ ^19^ ^]^ As shown in Figure 2c, the ratio of I_D1_/I_G_ and I_D3_/I_G_ of e_1_‐Fe‐N‐C exhibit the highest value (I_D1_/I_G_ = 1.91, I_D3_/I_G_ = 0.56) among e_x_‐Fe‐N‐C samples, and as the usage amount of zinc acetate decreases, the I_D1_/I_G_ and I_D3_/I_G_ decreases, unambiguously proving that the controllable modulation of the degree of defect in catalysts can be successfully achieved by regulating the amount of zinc acetate. X‐ray photoelectron spectroscopy (XPS) spectra were collected to determine the C–N environment for the further exploration of the defect degree in e_x_‐Fe‐N‐C catalysts. As seen in the high‐resolution C 1s spectra of the XPS, carbon species of e_x_‐Fe‐N‐C samples are divided into four types (Figure S8, Supporting Information), including C─C/C═C (≈284.2 eV), C–N (285.6 eV), C–O (286.7 eV), and C═O (288.3 eV). Geometrically, the edge‐located nitrogen atom can only bond with one carbon atom in a defect‐rich environment, whereas the centered nitrogen atom can simultaneously connect with two carbon atoms.^[^ ^13^ ^]^ Therefore, materials with more carbon defects should have fewer C─N bonds. As expected, e_1_‐Fe‐N‐C has the lowest proportion of C─N bonds compared to other samples, representing its highest content of edge‐located nitrogen atoms to bond with Fe (Figure 2d).

### 2.2 Atomic‐Scale Structure Analysis

**Figure 3:**
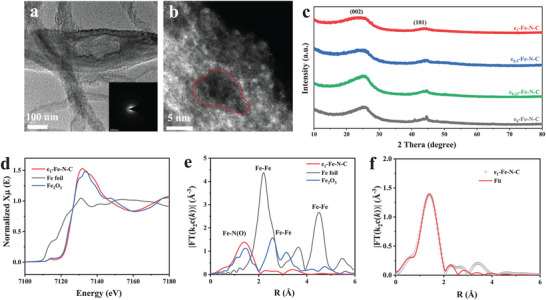
a) HRTEM (inset: the corresponding SAED pattern) and b) HAADF‐STEM images for e_1_‐Fe‐N‐C. c) XRD patterns of e_x_‐Fe‐N‐C. d) Normalized XANES spectra at the Fe K‐edge of Fe foil, Fe_2_O_3_, and e_1_‐Fe‐N‐C. e) FT‐EXAFS spectra of Fe K‐edge in sample R‐space. f) EXAFS fitting curves of e_1_‐Fe‐N‐C.

The e_1_‐Fe‐N‐C catalyst possessed a rich‐defect environment with the highest mesoporous/macropores specific surface area (793.29 m^2^ g^−1^), effectively preventing the aggregation of NPs and allowing for the loading of numerous edge‐located single‐atom active sites.^[^ ^20^ ^]^ As shown in **Figure** **3**a, HRTEM and the corresponding selected area electron diffraction (SAED) were employed to demonstrate the absence of NPs in the e_1_‐Fe‐N‐C. The aberration‐corrected high‐angle annular dark‐field scanning transmission electron microscopy (HAADF‐STEM) provides an insightful characterization of e_1_‐Fe‐N‐C, and the isolated bright dots throughout the carbon matrix represent the presence of atomic Fe sites (Figure 3b). Notably, these bright dots were densely distributed around the pores (marked with the red dashed lines), which preliminarily demonstrates that a large number of edge‐located atomic Fe sites have been generated via the present synthesis strategy. The X‐ray diffraction (XRD) result of e_1_‐Fe‐N‐C in Figure 3c can also verify the absence of crystalline Fe with only two broad diffraction peaks at 2θ = 24.1° and 43.8°, which correspond to the (002) and (101) planes of graphitic carbon, respectively.^[^ ^21^ ^]^ Besides, the presence of crystalline Fe (PDF no. 06–0696) in the e_0_‐Fe‐N‐C indicates the effectiveness of Zn in preventing metal aggregation, consistent with the HRTEM images of e_x_‐Fe‐N‐C in Figure 2a. The detailed chemical composition and electronic structure of the e_1_‐Fe‐N‐C catalyst were further disclosed by XPS (Figure S9, Supporting Information). The XPS survey scan confirms the presence of C, N, O, and Fe in e_1_‐Fe‐N‐C (Figure S9a, Supporting Information), which is consistent with the EDS mapping result (Figure S10, Supporting Information). The N species of e_1_‐Fe‐N‐C were quantitatively analyzed by the N 1s XPS spectra. The peaks appearing at 398.6, 399.8, 400.7, 401.5, and 403.9 eV are corresponding to pyridinic‐N, M‐N_X_ (N atoms bonded to metal sites), pyrrolic‐N, graphitic‐N, and oxidized‐N, respectively (Figure S9b, Supporting Information).^[^ ^22^ ^]^ As shown in Figure S11 (Supporting Information), the percentage of pyridinic‐N, M‐N_X_, and pyrrolic‐N are similar in the e_x_‐Fe‐N‐C samples (x = 1, 0.5, and 0.25), suggesting that the amount of introduced zinc acetate would not significantly alter the corresponding nitrogen configurations of the catalysts. These similar nitrogen configurations in the control samples precisely eliminate their significant effects on ORR performance, which is favorable for exploring the influence of the defect environment on ORR activity. The high‐resolution Fe 2p XPS spectrum of e_1_‐Fe‐N‐C was deconvoluted into Fe^3+^ peaks (726.2 and 712.1 eV), Fe^2+^ peaks (724.2 and 709.5 eV), and satellite peaks (719.4 and 715.2 eV) (Figure S9c, Supporting Information).^[^ ^23^ ^]^ No signal of metallic Fe was detected, which is consistent with the XRD pattern of e_1_‐Fe‐N‐C in Figure 3c. To further identify the chemical state and local coordination structure of Fe active sites in e_1_‐Fe‐N‐C, X‐ray absorption near‐edge structure (XANES) was conducted. As shown in Figure 3d, the energy absorption thresholds of e_1_‐Fe‐N‐C are close to that of Fe_2_O_3_ and far from Fe foil, demonstrating that the Fe atoms possess positive charges in e_1_‐Fe‐N‐C. The Fourier‐transformed extended X‐ray absorption fine structure (FT‐EXAFS) of the Fe K‐edge spectra in the R‐space is shown in Figure 3e.^[^ ^24^ ^]^ The prominent peak at ≈1.4 Å can be identified in the e_1_‐Fe‐N‐C, which is assigned to the first coordination shell of the Fe‐N(O) scattering path. Moreover, no related peak of the Fe‐Fe bond at ≈2.2 Å was detected, implying Fe in the e_1_‐Fe‐N‐C exists in the form of single atoms without the formation of any Fe NPs.^[^ ^25^ ^]^ The EXAFS curve‐fitting results reveal that it comprises four nitrogen atoms around Fe at 2.04 Å, confirming the configuration of edge‐located Fe‐N_4_ moieties in e_1_‐Fe‐N‐C (Figure 3f; Table S1, Supporting Information).

Based on the above discussion, we have highlighted the trends and causative factors for the changes in porous structure and defect content of e_x_‐Fe‐N‐C catalysts, as well as distinctly demonstrated the edge‐located Fe‐N_4_ in e_1_‐Fe‐N‐C. The Fe contents of different catalysts have been quantified by inductively coupled plasma‐mass spectrometry (ICP‐MS) and all four samples of the e_x_‐Fe‐N‐C catalysts have similar content of atomic Fe (Table S2, Supporting Information), which facilitates the correlation study between the degree of defect and ORR catalytic activities without the interference of other factors.

### 2.3 Electrocatalytic Activity Evaluation

**Figure 4:**
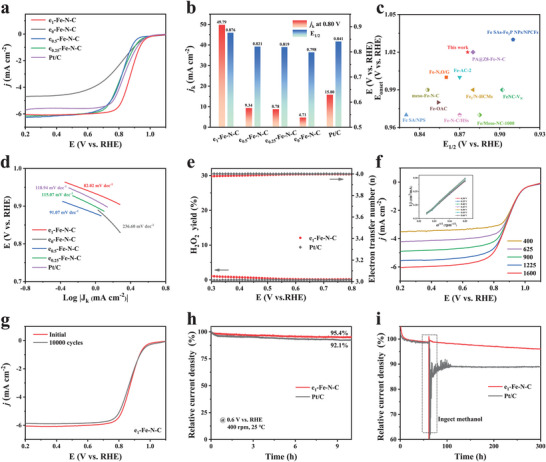
a) LSV curves of RRDE measurements at 1600 rpm in 0.1 m KOH. b) comparison of E_1/2_ and *j*_k_ at 0.8 V versus RHE for e_x_‐Fe‐N‐C and commercial Pt/C. c) Activity comparison of e_1_‐Fe‐N‐C with many previously reported catalysts. d) Tafel plots derived from L–S–V curves in (a). e) The calculated H_2_O_2_ selectivity and electron transfer number (*n*) for e_1_‐Fe‐N‐C and commercial Pt/C. f) L–S–V curves of e_1_‐Fe‐N‐C at various rotational speeds (inset: corresponding K–L plot). g) LSV curves of e_1_‐Fe‐N‐C before and after 10 000 potential cycles. h) The chronoamperometry test in O_2_‐saturated 0.1 m KOH at 0.6 V versus RHE with the rotation speed of 400 rpm for e_1_‐Fe‐N‐C and commercial Pt/C. i) Chronoamperometric curves of e_1_‐Fe‐N‐C and commercial Pt/C at 0.4 V versus RHE and 1600 rom with methanol addition at ≈60s.

The ORR activity of the as‐prepared e_x_‐Fe‐N‐C catalysts and other referenced samples were measured in O_2_‐saturated 0.1 m KOH electrolyte. The cyclic voltammetry (*C*–*V*) curve of e_1_‐Fe‐N‐C in the O_2_‐saturated solution shows a pronounced peak centered at 0.852 V versus RHE compared with that in the Ar‐saturated solution, providing a preliminary indication of the excellent ORR performance of e_1_‐Fe‐N‐C (Figure S12, Supporting Information).^[^ ^26^ ^]^ In Figure S13 (Supporting Information), more positive linear sweep voltammetry (LSV) polarization curves for e_1_‐Fe‐N‐C and other samples introduced with different Fe contents than that of the e_1_‐N‐C catalyst containing more defects (I_D1_/I_G_ = 1.95 and I_D3_/I_G_ = 0.67, Figure S6, Supporting Information) without the introduction of Fe, proving that the boosted ORR activity of e_x_‐Fe‐N‐C catalysts originates from the atomic Fe sites rather than N‐modified divacancies configuration. In addition, the secondary pyrolysis temperature for preparing e_1_‐Fe‐N‐C was optimized at 1000 °C due to the decline in ORR activity at either higher or lower temperatures (Figure S14, Supporting Information). As shown in **Figure** **4**a,b, e_1_‐Fe‐N‐C exhibits the E_1/2_ of 0.876 V and a kinetic current density (*j*_k_) of 49.79 mA cm^−2^ at 0.80 V, which surpass those of commercial Pt/C (E_1/2_ = 0.841 V, *j*_k_ = 15.80 mA cm^−2^) and other e_x_‐Fe‐N‐C catalysts (e_0.5_‐Fe‐N‐C: E_1/2_ = 0.821 V, *j*_k_ = 9.34 mA cm^−2^; e_0.25_‐Fe‐N‐C: E_1/2_ = 0.819 V, *j*_k_ = 8.78 mA cm^−2^; e_0_‐Fe‐N‐C: E_1/2_ = 0.798 V, *j*_k_ = 4.71 mA cm^−2^). Obviously, the ORR catalytic performance is significantly affected by the degree of defect in catalysts, and the higher degree of defects and edge‐located Fe‐N_4_ sites result in better ORR activity. Moreover, e_1_‐Fe‐N‐C features excellent ORR activity among similar catalysts (Figure 4c; Table S3, Supporting Information). Compared to e_x_‐Fe‐N‐C and commercial Pt/C catalysts, e_1_‐Fe‐N‐C shows the smallest Tafel slope of 82.02 mV dec^−1^, demonstrating its fast ORR kinetics (Figure 4d).^[^ ^27^ ^]^ The electron transfer number (*n*) and H_2_O_2_ yield were probed by the rotating ring‐disk electrode (RRDE) measurement. The electron transfer number ranges from 3.98 to 4.00 in the wide potential range from 0.30 to 0.80 V with the low yield of H_2_O_2_ (less than 1.5%), providing further evidence of the ideal dominant 4 e^−^ ORR pathway selectivity of e_1_‐Fe‐N‐C (Figure 4e), which are similar to those of commercial Pt/C. In addition, the value of *n* is also calculated from the L–S–V curves by rotating disk electrode (RDE) measurements under different rotating rates. As shown in Figure 4f, the fitted Koutecky–Levich (K–L) plot of e_1_‐Fe‐N‐C is well‐linear and the value of *n* is calculated to be ≈4, further revealing a typical 4e^−^ ORR pathway. The accelerated degradation test was carried out to illustrate the superior durability of e_1_‐Fe‐N‐C during the ORR. As seen in Figure 4g, e_1_‐Fe‐N‐C shows robust stability with a negligible decrease in E_1/2_ after continuous 10 000 CV cycles. The chronoamperometry test in O_2_‐saturated 0.1 m KOH at the rotation speed of 400 rpm further confirms the outstanding stability (Figure 4h). The e_1_‐Fe‐N‐C catalyst maintains 95.4% of the initial current density after a 10‐h continuous testing, which exceeds that of Pt/C (92.1%). Furthermore, the stability of the structure of e_1_‐Fe‐N‐C was also explored by HRTEM and Raman. As shown in Figure S15 (Supporting Information), the morphological structure of e_1_‐Fe‐N‐C remains almost unchanged without any NPs after the durability test, revealing the fundamental reason for its long‐lasting excellent catalytic performance. Besides, e_1_‐Fe‐N‐C displays a retained high current density after the injection of methanol, whereas the current of Pt/C drops significantly, verifying the superior methanol tolerance of e_1_‐Fe‐N‐C (Figure 4i).

### 2.4 Catalytic Mechanism by Theoretical Calculations

**Figure 5:**
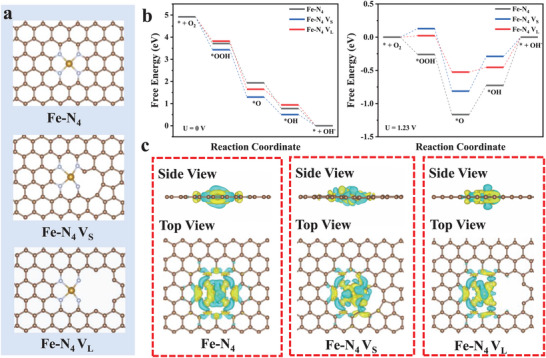
a) The optimized geometrical structures of Fe‐N_4_, Fe‐N_4_ V_S_, and Fe‐N_4_ V_L_ model. b) Free energy profiles of ORR on Fe‐N_4_, Fe‐N_4_ V_S_, and Fe‐N_4_ V_L_ at U = 0 V and U = 1.23 V. c) Top and side views of the computed differential charge density among Fe‐N_4_, Fe‐N_4_ V_S_, and Fe‐N_4_ V_L_.

To theoretically understand the origin of the high ORR activity of the e_1_‐Fe‐N‐C catalyst at the atomic level, density functional theory (DFT) calculations were performed on the defect‐induced electronic distribution asymmetry of Fe‐N_4_ sites and the corresponding catalytic ORR behaviors (details in the Supporting Information). To model the e_x_‐Fe‐N‐C catalysts with increasing carbon defects according to the amount of zinc acetate, edge‐located Fe‐N_4_ structures were constructed using small/large size of carbon vacancy next to the Fe‐N_4_ site, which are donated as Fe‐N_4_ V_S_ and Fe‐N_4_ V_L_, respectively. As shown in **Figure** **5**a, there is only one edge‐located nitrogen atom bonded with one carbon atom in the Fe‐N_4_ Vs structure, whereas there are two edge‐located nitrogen atoms bonded with one carbon atom in the Fe‐N_4_ V_L_ structure, which is consistent with the XPS results of e_x_‐Fe‐N‐C in Figure 2d. The free energy profiles of the ORR process on active sites of Fe‐N_4_, Fe‐N_4_ Vs, and Fe‐N_4_ V_L_ were calculated at zero bias and 1.23 V, and the structures of absorbed ^*^OOH, ^*^O, and ^*^OH on such different Fe‐N_4_ sites were exhibited in Figure 5b. All the steps are thermodynamically downslope at the ideal electrode potential for ORR (U = 0 V), proving a spontaneous exothermal process under this condition. However, upon increasing the potential to the equilibrium potential (U = 1.23 V), the structures of Fe‐N_4_, Fe‐N_4_ Vs, and Fe‐N_4_ V_L_ have their own highest energy barrier during the ORR process, which means the sluggish reaction dynamics at this equilibrium potential. As shown in Figure S16 (Supporting Information), the rate determine step (RDS) for ORR of typical Fe‐N_4_ was the last elementary step (^*^OH removal to form H_2_O), with an energy barrier of 0.73 eV. RDS for ORR of Fe‐N_4_ Vs was the third elementary step (^*^O reacts with one proton to form ^*^OH), with an energy barrier of 0.52 eV(Figure S17, Supporting Information). RDS for ORR of Fe‐N_4_ V_L_ was also the last elementary step, with an energy barrier of 0.45 eV (Figure S18, Supporting Information). Besides, the theoretical limiting potentials that reflect the ORR performance of Fe‐N_4_, Fe‐N_4_ Vs, and Fe‐N_4_ V_L_ are 0.5, 0.71, and 0.78 V, respectively. The lower energy barrier value and higher theoretical limiting potential for Fe‐N_4_ V_L_ than Fe‐N_4_ and Fe‐N_4_ Vs demonstrate that vacancy defect can effectively affect the ORR activity of nearby its neighboring Fe‐N_4_ site, and the large size carbon vacancy precedes the small size carbon vacancy in catalyzing ORR.

It's well known that too strong binding of the intermediates leads to poisoning of the catalyst surface while too weak binding leads to sluggish reactant activation, therefore, the excellent ORR activity of catalysts originates from the moderate bonding of their active sites to the ORR intermediates, which is extremely affected by the electronic structure of the active site.^[^ ^28^ ^]^ Differential charge density and Bader charge analysis were performed to investigate the spatial charge distribution at Fe‐N_4_, Fe‐N_4_ Vs, and Fe‐N_4_ V_L_ and shown in Figure 5c and Figure S19 (Supporting Information), where the yellow bubbles represent the charge accumulation and blue bubbles represent the charge depletion. Apparently, the introduction of adjacent carbon vacancy induced the electronic distribution asymmetry of Fe‐N_4_ sites. The Bader charge values of three models show that carbon vacancy inhibits adjacent N atoms from gaining electrons (+0.96 e^−^ for Fe‐N_4_ V_L_ and +0.97 e^−^ for Fe‐N_4_ V_S_), which is less than N atoms in Fe‐N_4_ (+1.21 e^−^). Furthermore, the C atom beside the vacancy in Fe‐N_4_ V_L_ loses more electrons (−0.59 e^−^) than Fe‐N_4_ V_S_ (−0.56 e^−^) and the C atom adjacent to N atom in Fe‐N_4_ (−0.46 e^−^), resulting in the accumulation of more negative charges near the Fe‐N site and the formation of partially reduced Fe state, thus reducing the energy barrier for ORR process of catalysts.^[^ ^10e^ ^]^ The unique electronic properties of Fe‐N_4_ V_L_ with electronic distribution asymmetry cause the polarized surface charges and appropriate adsorption/desorption capacity for oxygen intermediates. These results demonstrate that the introduced defects play a significant role in boosting ORR performance of SACs by modulating the electronic structure and binding affinities between oxygen intermediates and single‐atom active sites.

### 2.5 Zinc–Air Battery Performance

**Figure 6:**
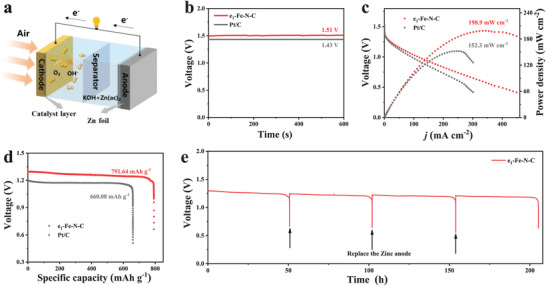
a) Schematic diagram of zinc–air battery (ZAB). b) Open circuit potential. c) Discharge polarization curves and power density plots. d) Voltage‐Specific capacity curves at 10 mA cm^−2^. e) Cycling tests of e_1_‐Fe‐N‐C‐based ZAB at 10 mA cm^−2^.

Inspired by the impressive ORR catalytic performance, a home‐made zinc–air battery (ZAB) armed with e_1_‐Fe‐N‐C on the air cathode was assembled to employ the potential of e_1_‐Fe‐N‐C in the practical application, and ZAB with Pt/C was adopted for comparison (**Figure** **6**a). The ZAB driven by e_1_‐Fe‐N‐C obtains a high open‐circuit voltage (OCV) of 1.51 V, which is significantly higher than that of the commercial Pt/C (1.43 V) and close to the theoretical value of 1.65 V, proving its higher output voltage (Figure 6b).^[^ ^29^ ^]^ Discharge polarization curves and the corresponding power density of catalysts are plotted in Figure 6c, and the e_1_‐Fe‐N‐C‐based ZAB delivers a maximum power density of 198.9 mW cm^−2^ at the current density of 340.0 mA cm^−2^, which surpasses that using commercial Pt/C (152.3 mW cm^−2^ at the current density of 249.3 mA cm^−2^). This is attributed to the excellent mass and charge transfer of the N‐doped porous carbon matrix with the edge‐located Fe‐N_4_ sites.^[^ ^30^ ^]^ Meanwhile, the specific capacity of the e_1_‐Fe‐N‐C‐based ZAB was calculated to be 791.64 mAh g^−1^, which is also much higher than that using the commercial Pt/C‐based battery (660.08 mAh g^−1^) (Figure 6d). Moreover, due to its stable structure, the e_1_‐Fe‐N‐C‐based ZAB exhibits outstanding stability during the long‐time discharge. As seen in Figure 6e, negligible discharging voltage loss can be observed at the current density of 10 mA cm^−2^ even over 200 h. As a result, e_1_‐Fe‐N‐C shows excellent potential as a power source for future electronics.

## 3 Conclusion

In summary, the in situ zinc acetate assisted thermal etching strategy has been successfully employed to obtain a promising single‐atom ORR catalyst (e_1_‐Fe‐N‐C) enriched with edge‐located Fe‐N_4_ active sites. The amount of zinc acetate precursor can modulate the geometrical structure and the degree of defects in the carbon matrix, which ensures the edge‐located configuration of active metal centers of the as‐prepared catalyst. With the increasing amount of zinc acetate, mesopores and macropores structures that favor mass transfer and exposure of active sites are significantly increased. This process introduces more defects and edge‐located Fe‐N_4_ active sites into the catalyst. The edge‐located Fe‐N_4_ active sites endowed e_1_‐Fe‐N‐C excellent ORR activity in alkaline media (E_1/2_ = 0.876 V in 0.1 m KOH), making it promising for ZAB application (198.9 mW cm^−2^ at 340.0 mA cm^−2^). This work addresses the challenge of controllable synthesis of edge‐located active sites in conventional M‐N‐C catalysts and provides a new avenue for designing cost‐effective and efficient future electrocatalysts.

## Conflict of Interest

The authors declare no conflict of interest.

## Supporting information

Supporting Information

## Data Availability

The data that support the findings of this study are available from the corresponding author upon reasonable request.
