# Supplementary material for: Zinc Assisted Thermal Etching for Rich Edge‐Located Fe‐N4 Active Sites in Defective Carbon Nanofiber for Activity Enhancement of Oxygen Electroreduction
Source: Adv Sci (Weinh). 2024 Aug 19;11(39):2407294. doi: 10.1002/advs.202407294 (PMC11496982; doi:10.1002/advs.202407294)
Supplement: Supplementary file 1 — Supporting Information [file ADVS-11-2407294-s001.docx]

Supporting Information

Zinc Assisted Thermal Etching for Rich Edge-Located Fe-N_4_ Active Sites in Defective Carbon Nanofiber for Activity Enhancement of Oxygen Electroreduction

Ruoyu Pang, Hongyin Xia, Xieyiming Dong, Qian Zeng, Jing Li,* and Erkang Wang*

Ruoyu Pang, Hongyin Xia, Xieyiming Dong, Qian Zeng, Jing Li, Erkang Wang

State Key Laboratory of Electroanalytical Chemistry

Changchun Institute of Applied Chemistry, Chinese Academy of Sciences

Changchun 130022, China
*E-mail: lijingce@ciac.ac.cn; ekwang@ciac.ac.cn

Ruoyu Pang, Xieyiming Dong, Qian Zeng, Jing Li, Erkang Wang
School of Applied Chemistry and Engineering

University of Science and Technology of China

Hefei 230026, China

Keywords: Single-atom catalysts, Oxygen reduction reaction, Defect engineering, Electronic structure, Zinc-air battery.

**1.Experimental Procedures**

**1.1. Chemicals and reagents**

Zinc acetate dihydrate (Zn(CH_3_COO)_2_·2H_2_O) was acquired from Xilong Chemical Co., Ltd. Hemin, *N, N*-dimethylformamide (DMF), and polyacrylonitrile (PAN, M_w_ = 150000 g mol^-1^) were purchased from Macklin. Sulfuric acid (H_2_SO_4_), potassium hydroxide (KOH), and ethanol were obtained from Beijing Chemical Reagent (Beijing, China). Platinum on graphitized carbon catalyst (Pt/C, 20.0 wt % loading) and Nafion (5.0 wt %) were acquired from Sigma-Aldrich. All chemicals were of analytic grade and used without further purification. The deionized water (DI, 18.2 MΩ cm^-1^) purified by a Milli-Q system (Millipore, Bedford, MA) was used to prepare all the solutions.

**1.2. Synthesis of electrocatalysts**

The e_1_-Fe-N-C catalyst was synthesized by electrospinning technology with the zinc acetate thermal etching strategy. Typically, 0.5 g of PAN, 1 g of Zn(CH_3_COO)_2_·2H_2_O, and 65.2 mg of hemin were dissolved into 5 mL of DMF under magnetic stirring for 12 h at room temperature. Subsequently, the obtained homogeneous precursor solution was charged into a 5 mL plastic syringe with a stainless needle (19 #) to fabricate the raw PAN-based nanofiber membranes. The parameters of the electrospinning process are as follows: applied voltage of 15 kV, feed rate of 0.3 mm min^-1^, and spinneret-to-collector distance of 20 cm. Afterward, the obtained electrospun nanofiber membranes were pre-oxidized at 180 ℃ for 6 h in an air atmosphere followed by a two-stage calcination process in a tube furnace, in which the membranes were firstly annealed at 500 °C for 2 h with the heating rate of 5 °C min^-1^ in an NH_3_ atmosphere and then directly annealed up to 1000 °C with the heating rate of 5 °C min^-1^ for 2h under a Ar atmosphere. Finally, the product was soaked in 0.5 M H_2_SO_4_ solution overnight at 60 °C to remove the unstable species and washed with DI several times, and then dried at 80 °C overnight to generate e_1_-Fe-N-C. Besides, three contrastive samples were also synthesized using the above standard protocol except for adjusting the amount of Zn(CH_3_COO)_2_·2H_2_O (x = 0, 0.25, and 0.5g) and hemin (y = 32.6, 130.4 and 195.6 mg), and denoted as e_x_-Fe-N-C and e_1_-Fe-N-C-y, respectively. In addition, e_1_-N-C was also prepared by the same synthesis process of e_1_-Fe-N-C only without the addition of hemin.

**1.3. Materials characterization**

The morphology and detailed microstructures were recorded by scanning electron microscopy (SEM, ZEISS MERLIN Compact, 10kV), transmission electron microscopy (TEM, JEM-2100F), high-resolution TEM (HRTEM, JEOL JEM 2100plus), and aberration-corrected high-angle annular dark-field scanning transmission electron microscopy (HAADF-STEM, Titan Themsis Z). The crystal structural information and degree of graphitization were determined by X-ray powder diffraction (XRD, Bruker D8 Advance diffractometer with Cu Kα (λ= 1.5418 Å)) and Raman spectra (Renishaw 2000 model confocal microscopy Raman spectrometer with a CCD detector and a holographic notch filter). The specific surface area of the catalyst based on the Brunauer-Emmett-Teller (BET) model was calculated from the N_2_ adsorption/desorption isotherms measured with an ASAP 2020 Physisorption Analyzer. The chemical state of the material was explored by X-ray photoelectron spectroscopy (XPS) using an ESCALAB-MKII X-ray photoelectron spectrometer with Al Kα radiation. The X-ray absorption fine structure (XAFS) measurements at Fe K-edge under transmission mode were collected on Super Photon ring-8 in Harima Science Garden City, Hyogo. Inductively coupled plasma-mass spectrometry (ICP-MS) was used to analyze the metal contents of materials on a ThermoScientific CAP6300.

**1.4. Electrochemical measurement**

The oxygen reduction reaction (ORR) performance was employed on the CHI 832C electrochemical workstation in a 0.1 M KOH solution using a three three-electrode system. For the three-electrode configuration, the catalyst-modified glassy carbon rotating disk electrode (RDE, with a disk area of 0.19625 cm^2^) or rotating ring-disk electrode (RRDE, with a disk area of 0.2475 cm^2^ and a Pt ring area of 0.1866 cm^2^) as the working electrode, the graphite rod as the counter electrode and a Hg/HgO electrode (1 M KOH) as the reference electrode. All potentials reported were calibrated to the reversible hydrogen electrode (RHE) scale based on the following equation: E(RHE) = E(Hg/HgO) + 0.865 V.

The catalyst inks were prepared by dispersing 5 mg of catalyst in 980 μL of ethanol and 20 μL of 5 wt. % Nafion solution under sonication for 60 min. Subsequently, 10 μL of catalyst ink was drop-casted onto the freshly polished RDE and dried at room temperature with the mass loading of the catalyst was approximately 0.255 mg cm^-2^. All control samples were dropped on the electrode with the same loading.

Oxygen was bubbled in the 0.1 M KOH solution for 30 min to form an O_2_-saturated electrolyte before the ORR test. The catalysts were first active by conducting cyclic voltammetry (CV) between 0.2 and 1.1 V (*vs*. RHE) at a scan rate of 100 mV s^-1^. Linear sweep voltammetry (LSV) was conducted to evaluate the ORR performance at a scan rate of 10 mV s^-1^ with an applied potential range from 1.10 to 0.2 V (*vs*. RHE) under the rotating speed of 1600 rpm. The CV measurement for ORR performance was conducted at a scan rate of 20 mV/s. The accelerated degradation test by cycling between 0.2 V to 1.1 V at 50 mV s^-1^ for 10000 cycles. The chronoamperometry was conducted at a potential of 0.6 V (*vs*. RHE) at room temperature with a rotating speed of 400 rpm.

The electron transfer number (*n*) can be calculated according to the Koutecky-Levich (K-L) equation as follows:

$$\frac{\text{1}}{\text{j}}\text{ = }\frac{\text{1}}{\text{j}_{\text{l}}}\text{ + }\frac{\text{1}}{\text{j}_{\text{k}}}\text{=}\frac{\text{1}}{\text{B}\text{ω}^{\text{1/2}}}\text{ + }\frac{\text{1}}{\text{j}_{\text{k}}\text{}\text{}}\text{ }\text{ }\text{ }\text{ }\text{ }\text{ }\text{ }\text{ }\text{ (1)}$$

$$\text{B}\text{ = }{\text{0.2}\text{nFC}_{\text{0}}\text{D}}_{\text{0}}^{\text{2/3}}\text{υ}^{\text{-1/6}} \text{ (2)}$$

in which *j* is the experimental disk current density, *j*_l_ is the diffusion-limiting current density, *j*_k_ is the kinetic current density, $\text{ω}$ is the angular rotation speed of the working electrode in rad s^-1^, *F* is faraday constant (96,485 C mol^-1^), *C*_0_ is the bulk concentration of O_2_ (1.2×10^−6^ mol cm^−3^), *D*_0_ is the diffusion coefficient of O_2 ­_in 0.1 M KOH solution (1.9×10^−5^ cm^2^ s^−1^), and *υ* is the kinetic viscosity of 0.1 M KOH solution (0.01 cm^2^ s^−1^). The K-L plots can be derived from LSV curves at different rotation speeds (400, 625, 900, 1225, 1600 rpm) under various potentials (0.30, 0.35, 0.40, 0.45, 0.50, 0.55, 0.60 V).

In the RRDE experiments, 12.6 μL of ink was dropped on the RRDE and the potential of Pt-ring was set at 1.3 V (*vs*. RHE) to estimate the yield of hydrogen peroxide (H_2_O_2_) during ORR. Calculation of the yield of H_2_O_2_ and *n* are illustrated as follows:

$$\text{H}_{\text{2}}\text{O}_{\text{2}}\text{ }\text{\%}\text{ = }\frac{\text{I}_{\text{r}}\text{ /}{\text{ }\text{N}}_{\text{0}}}{\text{I}_{\text{d}}\text{ + }\text{I}_{\text{r}}\text{ / }\text{N}_{\text{0}}}\text{ × 200 }\text{ }\text{ (3)}$$

$$\text{n}\text{ = 4 ×}\frac{\text{I}_{\text{d}}}{\text{I}_{\text{d}}\text{ + }\text{I}_{\text{r}}\text{ / }\text{N}_{\text{0}}} \text{ (}\text{4}\text{)}$$

where *I*_r_ is the ring current, *I*_d_ is the disk current, and *N* = 0.37 is the current collection efficiency of the Pt ring in the RRDE.

Tafel slopes were calucated according to Tafel equation:

$$\text{η = b}\log(\text{j}/{\text{j}_{\text{0}})} \text{ (5)}$$

where $\text{η}$ is the overpotential ($\text{η }\text{=}\text{ }\text{|}\text{E}_{\text{RHE}}-1.23 V\text{|}$), $\text{b}$ is Tafel slope, $\text{j}$ is the experimental current density, and $\text{j}_{\text{0}}$ is the exchange current density.

**1.5. Computational details**

All the calculations are performed in the framework of the density functional theory with the projector augmented plane-wave method, as implemented in the Vienna ab initio simulation package.^[1]^ Spin polarization was also included. The generalzied gradient approximation (GGA) proposed by Perdew, Burke, and Ernzerhof is selected for the exchange-correlation potential.^[2]^ A DFT-D3 scheme of dispersion correction was used to describe the van der Waals (vdW) interactions in molecule adsorption.^[3]^ The cut-off energy for plane wave is set to 450 eV. The energy criterion is set to 1E-05 eV in iterative solution of the Kohn-Sham equation. The electron smearing width of σ = 0.03 eV was employed according to the Gaussian smearing technique. The Brillouin zone integration is performed using the uniformly distributed scattering of going through the Gamma point to select a 2x2x1 k-mesh in the Monkhorst-Pack grid to make structure optimization.^[4]^ All the structures are relaxed until the residual forces on the atoms have declined to less than 0.02 eV/Å.

The pathway by which the *+O_2_→*+ OH^-^ occurs under alkaline condition are generally reported to proceed according to the following step:

* + O_2_ + H_2_O + e^-^ → *OOH + OH^-^

*OOH + H_2_O + e^-^ → *O + OH^-^

*O + H_2_O + e^-^ → *OH + OH^-^

*OH + H_2_O + e^-^ → * + OH^-^

Where the * refers to the catalytic, and the *one refers to the species that adsorbed on the activity sites.

Neglect PV contribution to translation for adsorbed molecules, the free energy was calculated according to the equation of G = E + H_cor_ – TS = E + G_cor_, where E is the energy of every specie obtained from DFT calculations, and S are entropy, while T is 298.15 K. The H_cor_ and G_cor_ are the thermal correction to enthalpy and the thermal correction to Gibbs free energy, respectively. These G_cor_ of adsorbate* was taken from the frequency DFT calculation and got value by using Vaspkit.1.4.1.^[5]^ The Gibbs free energy of the proton-electron pairs related in the PECT progress,^[6]^ whereas the fact that the proton-electron pairs is in equilibrium with gaseous H_2_: G(H^+^ + e^-^) = 1/2 G(H_2_ (g)). According to Vaspkit.1.4.1, the internal energy of gas molecular gained from the formula: U(T) = ZPE + ΔU(0-T), the enthalpy of gas molecular gained from the formula: H(T) = U(T) + PV = ZPE + ΔU(0-T) + PV, and the Gibbs free energy of gas molecular gained from the formula: G(T) = H(T) - TS = ZPE + ΔU(0-T) + PV – TS = E_DFT + G_cor’. Where E_DFT is the energy of the free gas molecule obtained from DFT calculations, G_cor’ is the thermal correction to Gibbs free energy of the free gas molecule obtained from the frequency DFT calculation and got value by using Vaspkit.1.4.1, with the temperature of 298.15K, the pressure of H_2_(g) and H_2_O(l) were 1 atm and 0.035 bar, and H_2_, H_2_O input 1 as the value of spin multiplicity. Note that the free energy of a O_2_ gas molecule should be calculated by this equation: G (O_2_, g) = 2*G (H_2_O, l) – 2*G (H_2_, g) + 4.92 eV. The charge density difference was evaluated using the formula Δρ = ρ(1+2) - ρ(1) - ρ(2), then analyzed by using the VESTA code.^[7]^

**1.6. Fabrication and test of Zn-air batteries (ZABs)**

The ZABs tests were performed in a home-made Zn-air cell. The carbon cloth coated with catalyst of e_1_-Fe-N-C or commercial Pt/C (catalyst loading was 1 mg cm^-2^) was used as the air cathode and a polished Zn plate was used as the anode. An 6 M KOH + 0.2 M Zn(CH_3_COO)_2_ mixed solution was used as the electrolyte. The specific capacity and stability of battery were tested at the current density of 10 mA cm^-2^, and the specific capacity is calculated according to the following equation:

$$\text{specific}\text{ }\text{capacit}\text{y = }\frac{\text{current }\text{×}\text{ service hours}}{\text{weight of consumed zinc}} \text{ (6)}$$

All the batteries tests were performed on the CHI660D electrochemical workstation at the room temperature.

**2.Supplementary Figures and Tables**

**
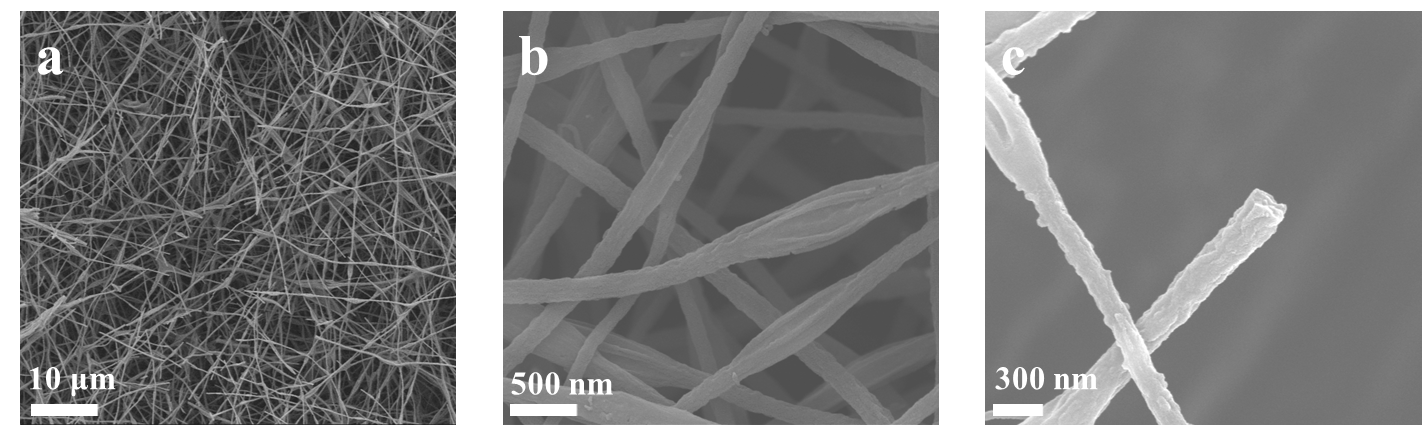
**

**Figure S1.** The SEM images of e_1_-Fe-N-C.


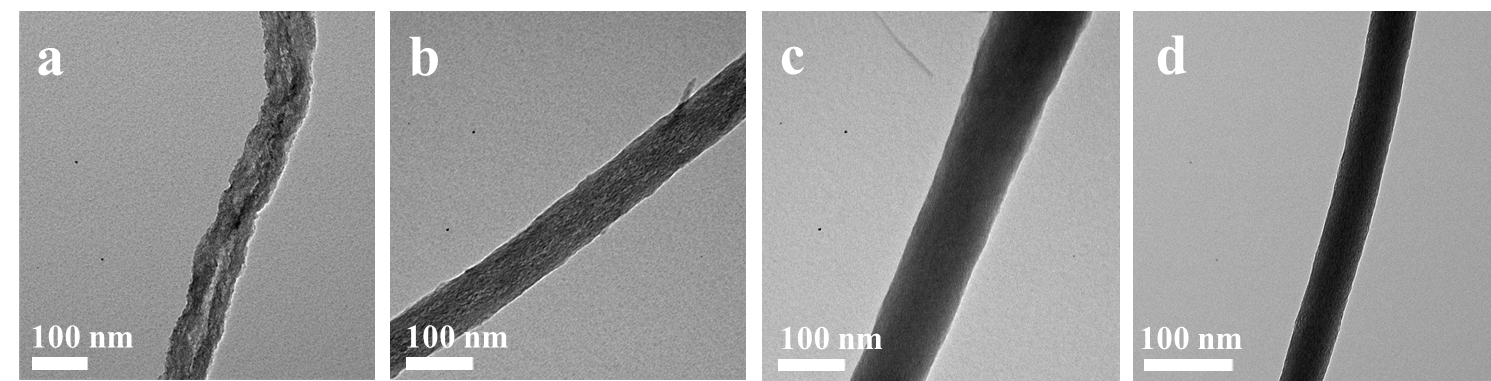


**Figure S2.** TEM images of e_x_-Fe-N-C catalysts. a) e_1_-Fe-N-C, b) e_0.5_-Fe-N-C, c) e_0.25_-Fe-N-C, and d) e_0_-Fe-N-C.

**
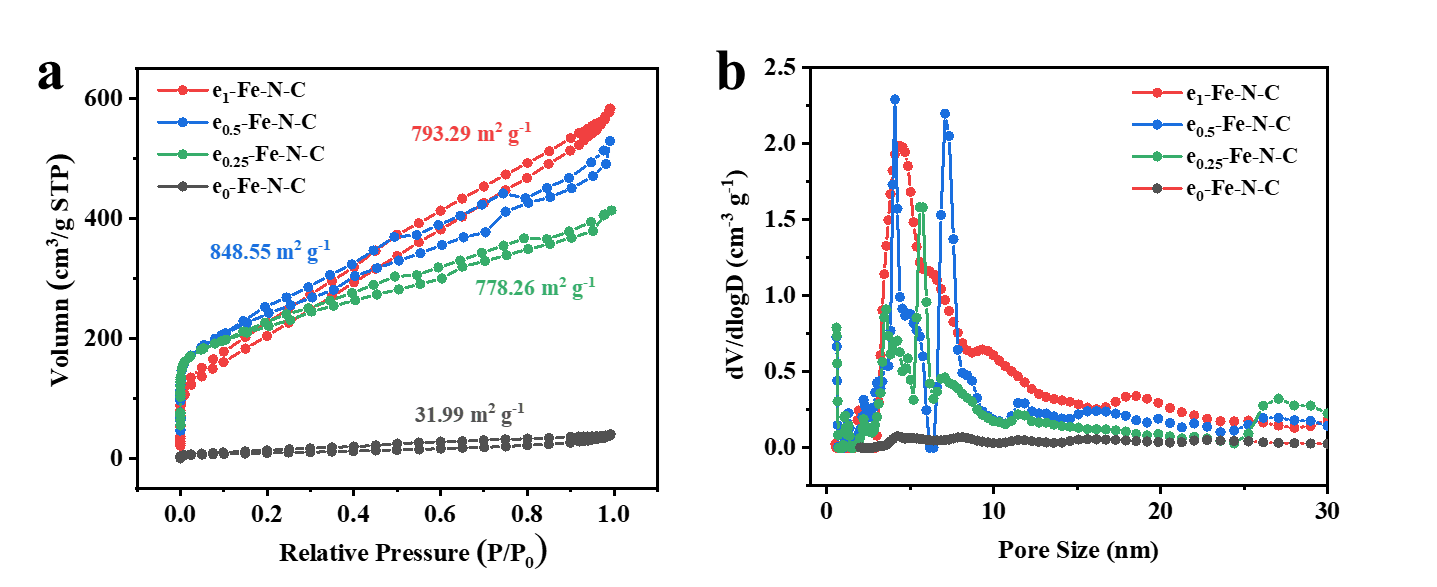
**

**Figure S3.** a) N_2_ adsorption and desorption isotherms and b) The corresponding pore size distribution curves of e_x_-Fe-N-C.

**
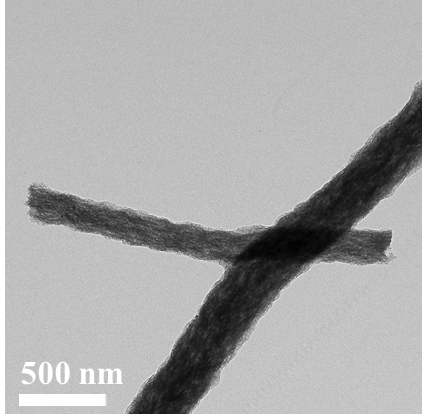
**

**Figure S4.** TEM image of e_1_-N-C.

**
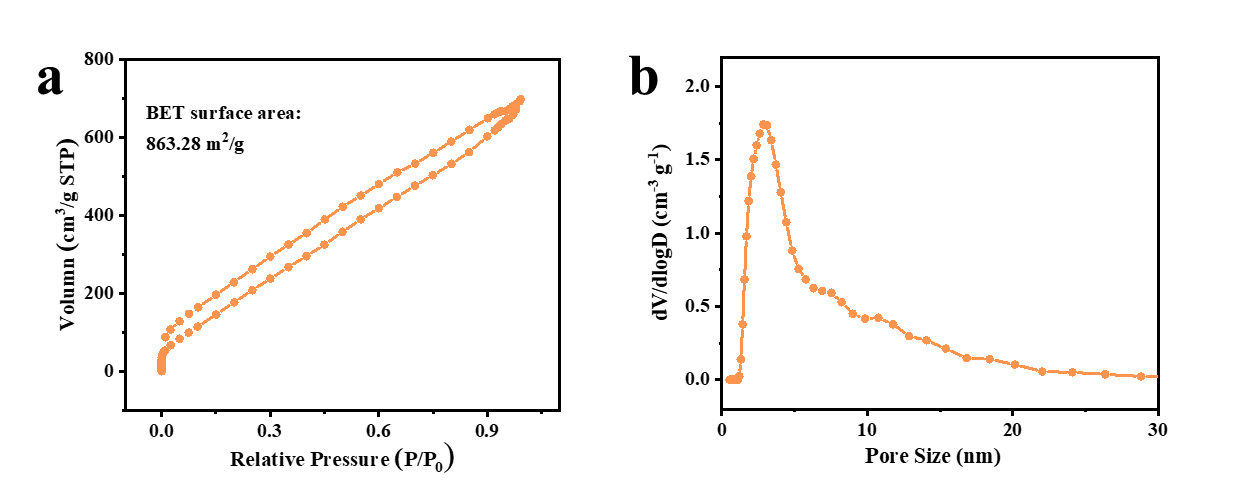
**

**Figure S5.** a) N_2_ adsorption and desorption isotherms and b) The corresponding pore size distribution curve of e_1_-N-C.

**Figure S6.** Raman spectra for the comparison of e_1_-Fe-N-C and e_1_- N-C.

**Figure S7.** Raman spectra for e_x_-Fe-N-C.

**Figure S8.** High-resolution C 1s XPS spectra of e_x_-Fe-N-C.

**
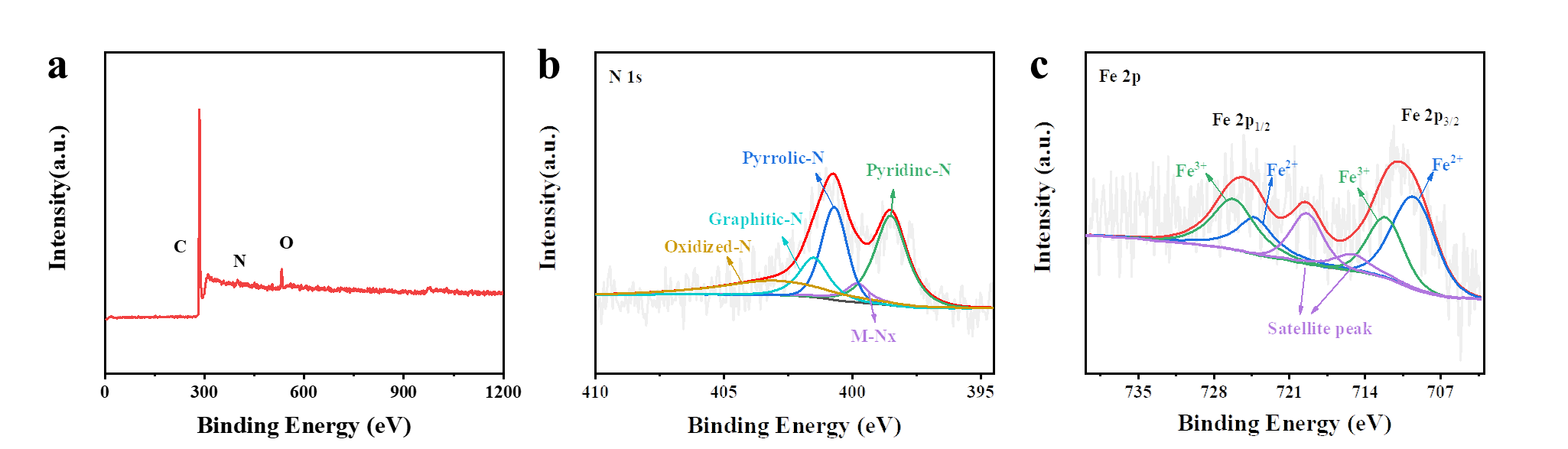
**

**Figure S9.** a) XPS survey scan, b) High-resolution N 1s XPS spectra, and c) High-resolution Fe 2p XPS spectra of e_1_-Fe-N-C.


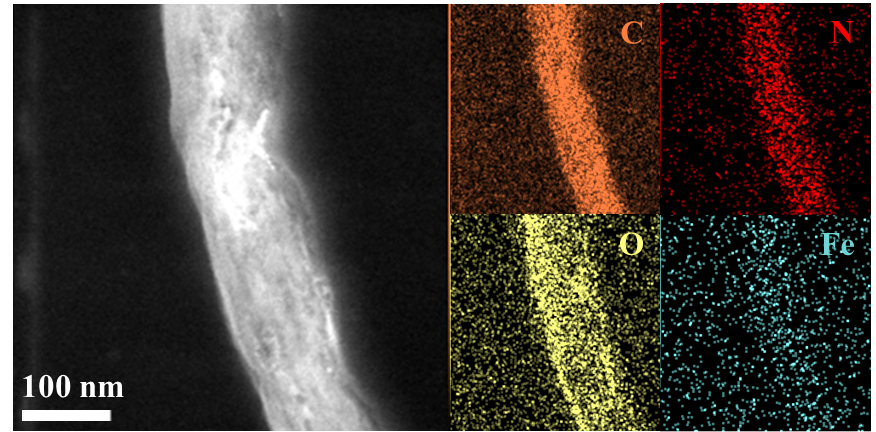


**Figure S10.** Elemental mapping (C, N, O, Fe) images for e_1_-Fe-N-C.

**
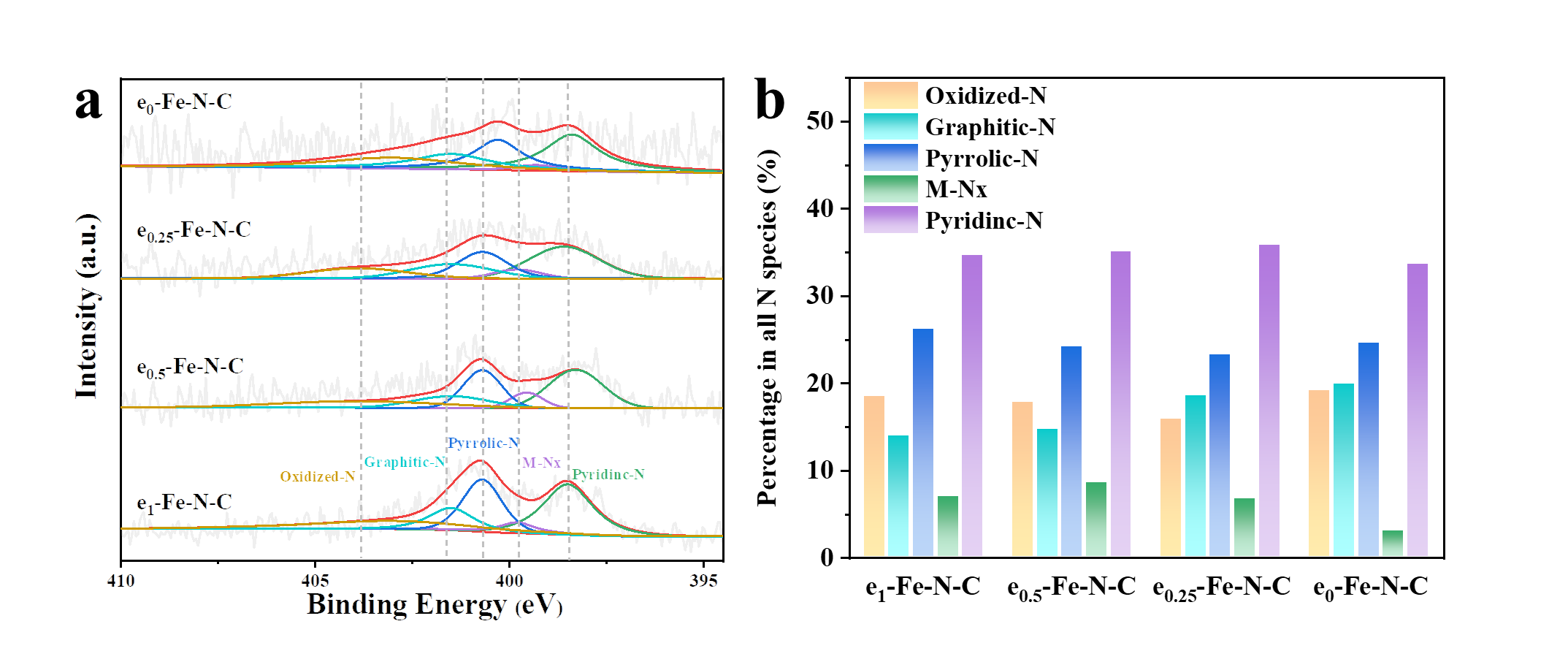
**

**Figure S11.** a) High-resolution N 1s XPS spectra and b) Summarized nitrogen contents of e_1_-Fe-N-C, e_0.5_-Fe-N-C, e_0.25_-Fe-N-C and e_0_-Fe-N-C.

**Figure S12.** CV curves of e_1_-Fe-N-C in Ar and O_2_ saturated 0.1 M KOH.

**Figure S13.** LSV curves of e_1_-Fe-N-C-y and other samples introduced with different contents of hemin (0 mg, 32.5 mg, 65.2 mg, 130.4 mg, and 195.6 mg) in O_2_-saturated 0.1 M KOH.

**Figure S14.** LSV curves of e_1_-Fe-N-C with different secondary pyrolysis temperature (850 ℃, 900 ℃, 1000 ℃, and 1100 ℃) in O_2_ saturated 0.1 M KOH.

**
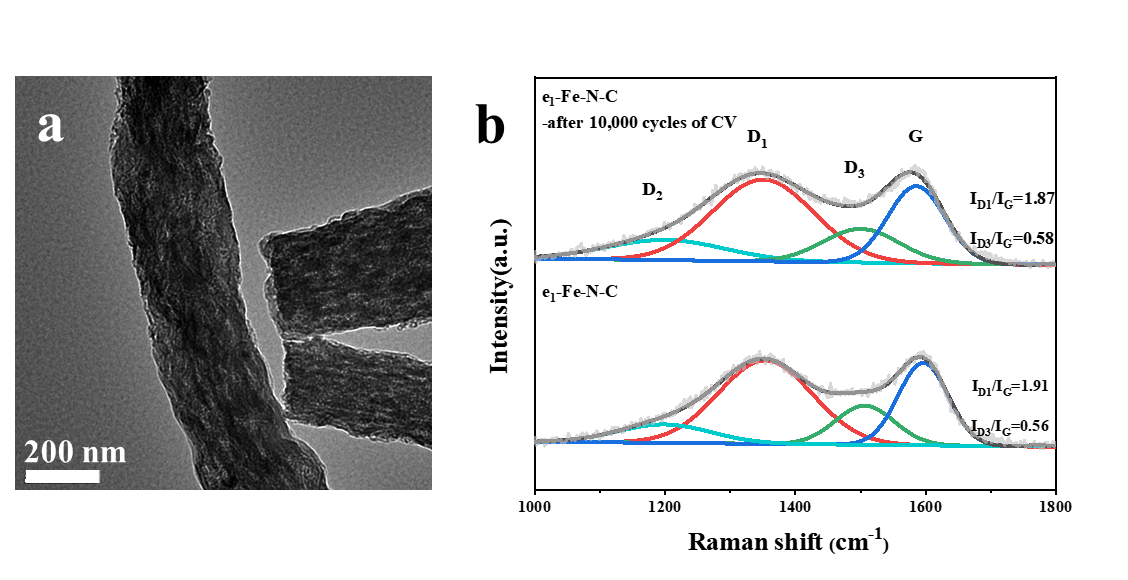
**

**Figure S15.** a) HRTEM and b) Raman of e_1_-Fe-N-C after stability test.


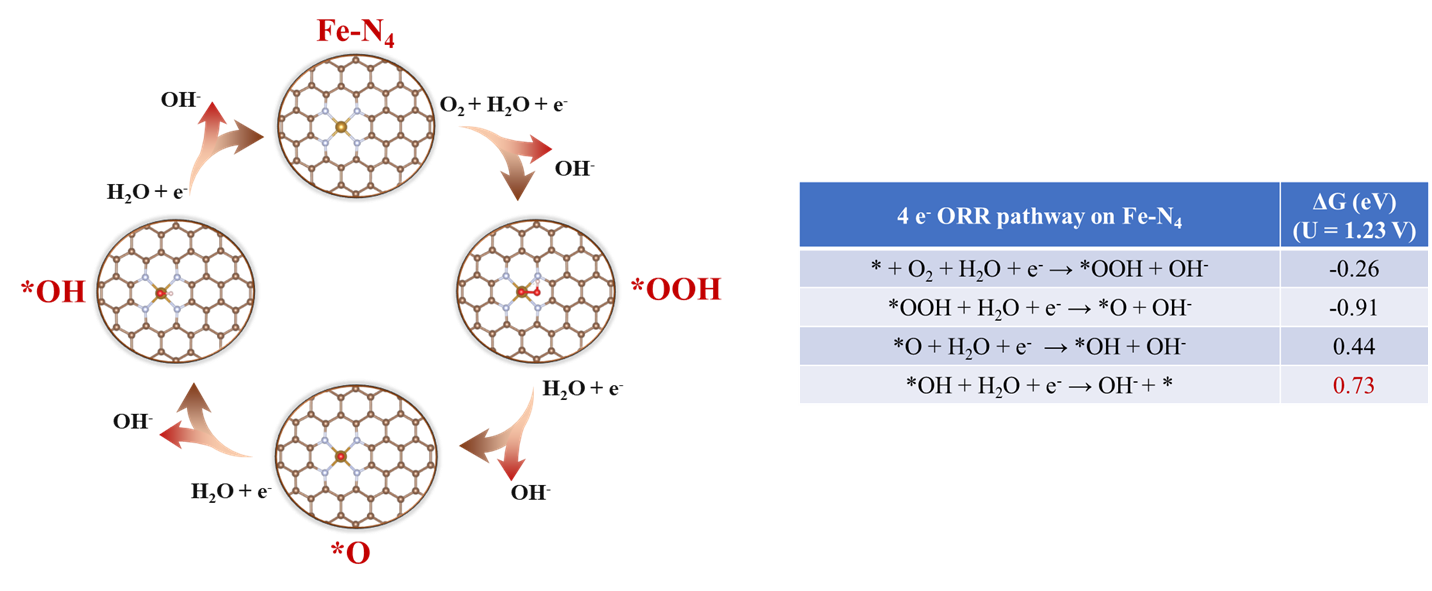


**Figure S16.** a) The optimized structure of ORR intermediates on the Fe–N_4_ model and the corresponding calculated free energy.


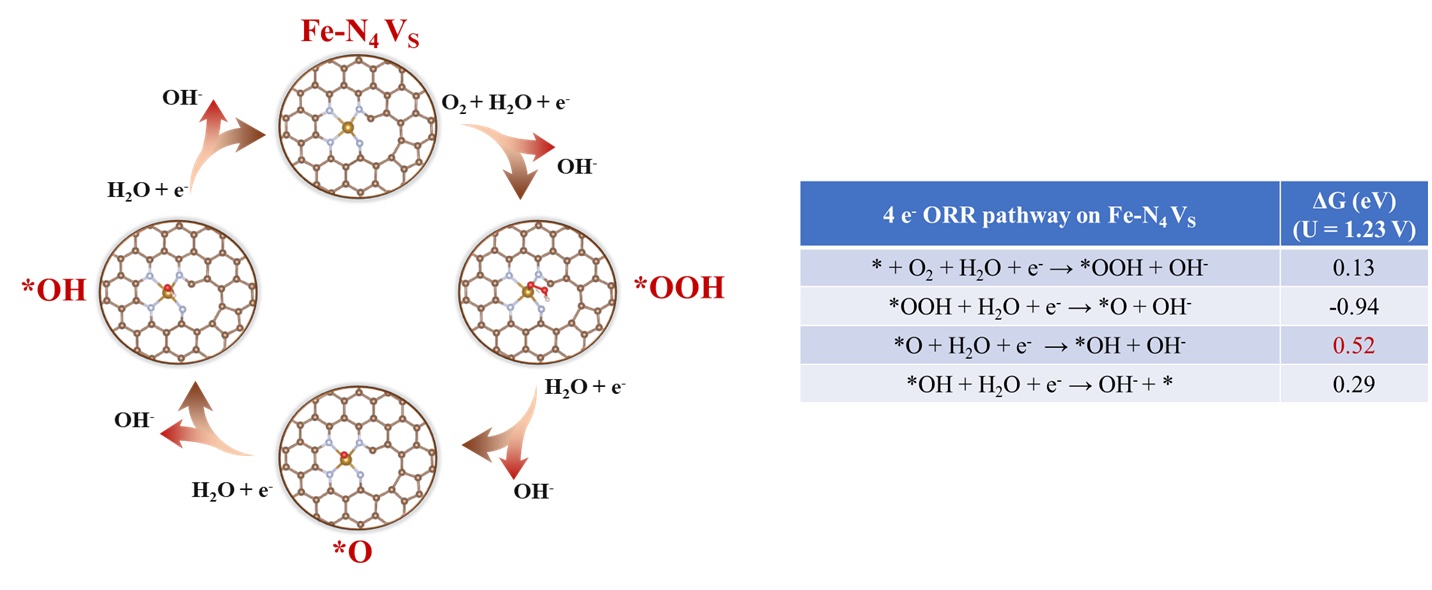


**Figure S17.** a) The optimized structure of ORR intermediates on the Fe–N_4_ V_S_ model and the corresponding calculated free energy.


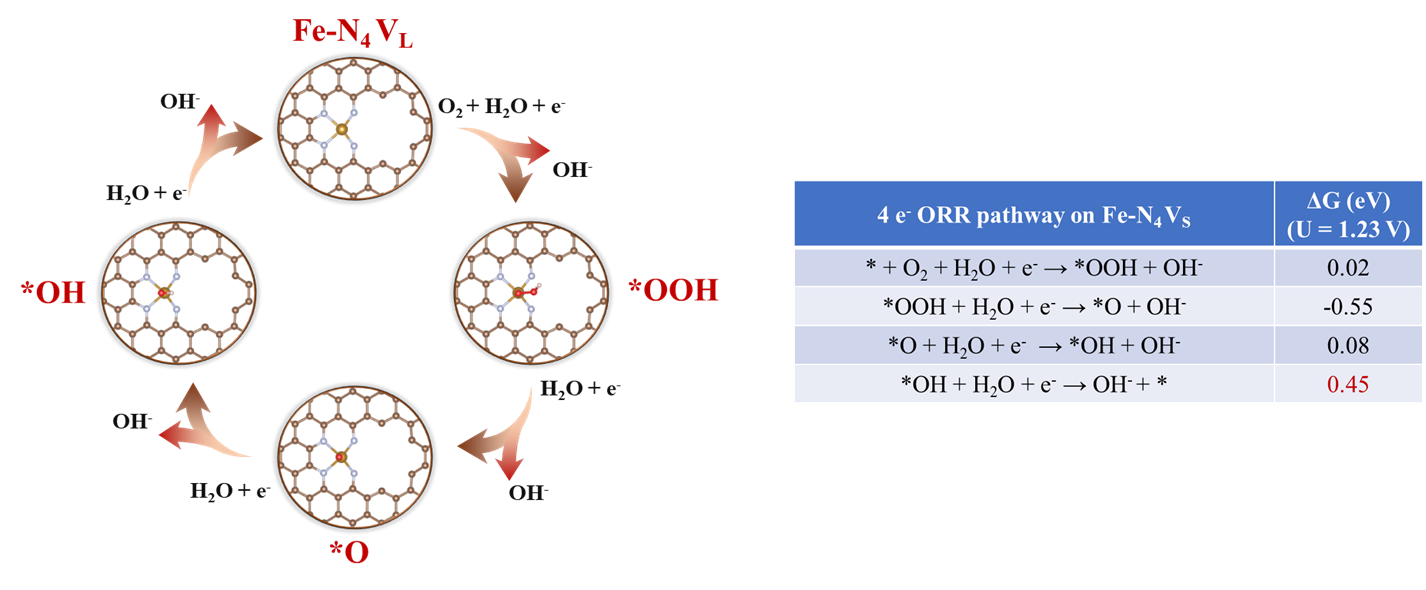


**Figure S18.** a) The optimized structure of ORR intermediates on the Fe–N_4_ V_L_ model and the corresponding calculated free energy.


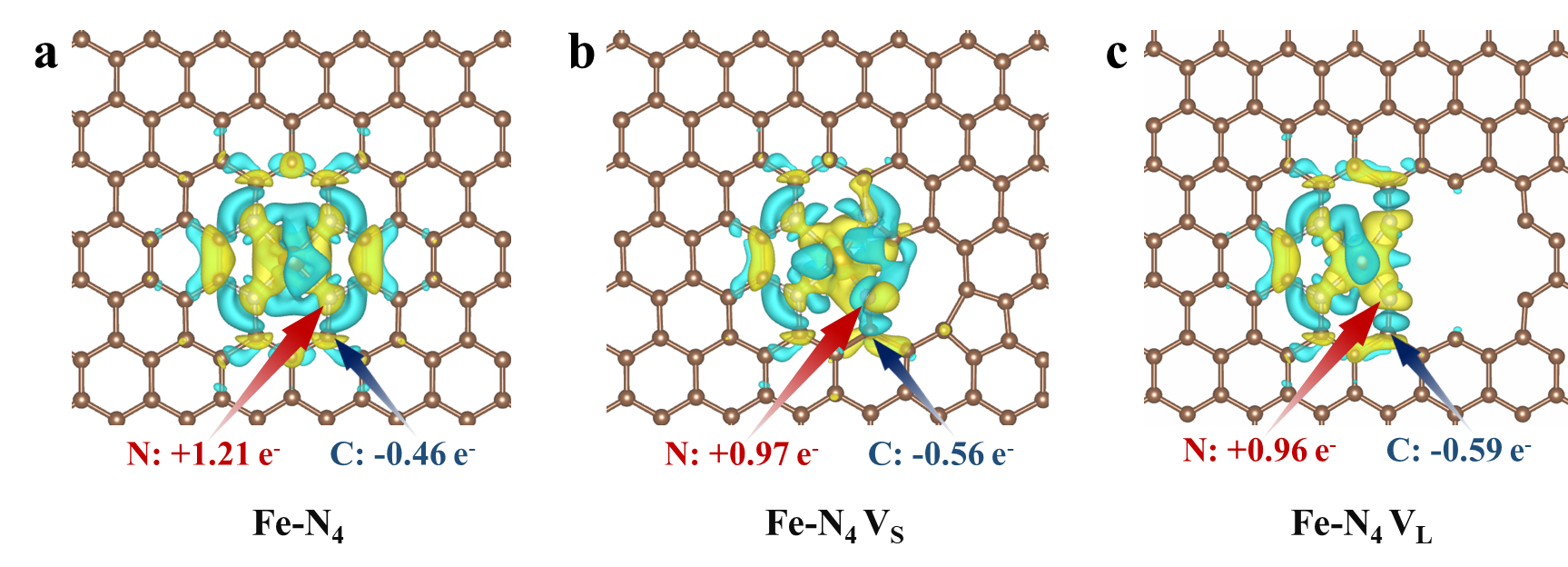


**Figure S19.** Charge density difference diagram and Bader charge for a) Fe–N_4_, b) Fe–N_4_ V_S_, and c) Fe–N_4_ V_S_.

**Table S1.** EXAFS data fitting results of Fe-SA-PNC.


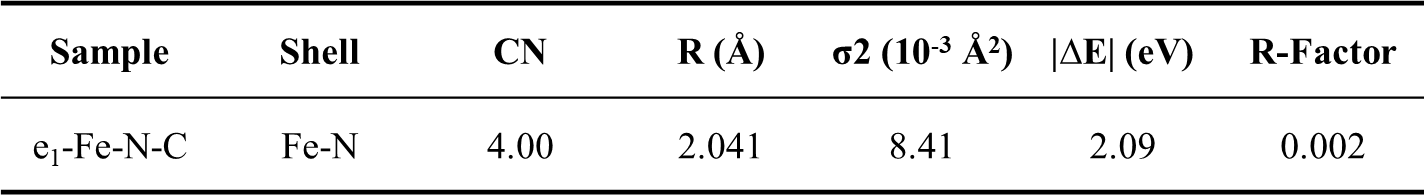


CN, coordination number; R, distance between absorber and backscatter atoms; σ^2^ Debye-Waller factor; ∆E, inner potential correction; R-Factor is used to value the goodness of the fitting.

**Table S2.** The content of the elements in the prepared e_1_-Fe-N-C.


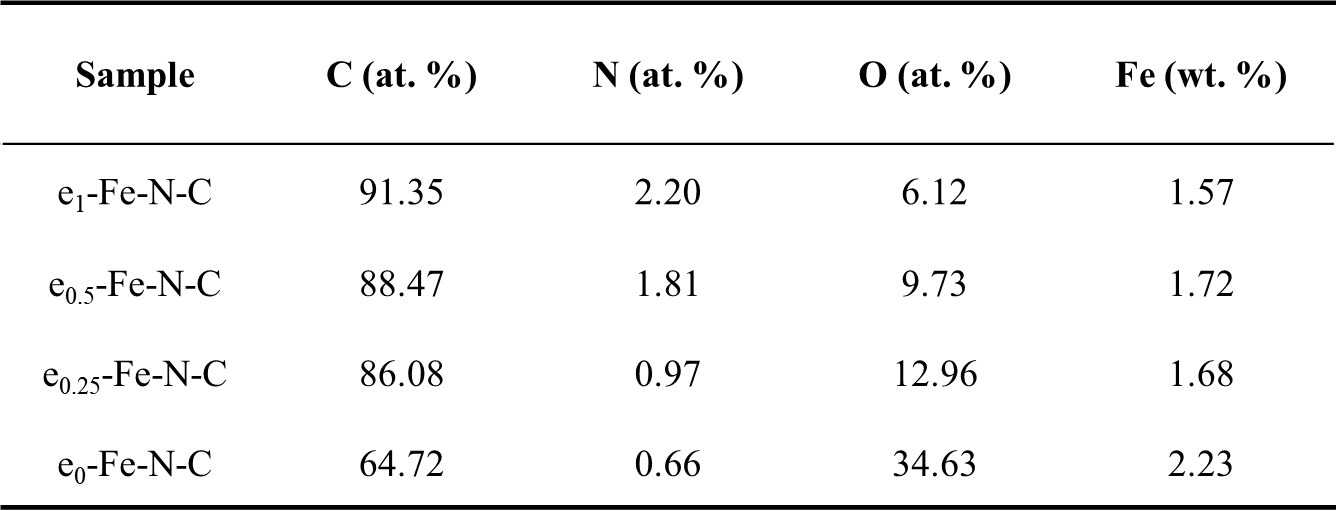


The content of Fe is determined by ICP-MS, and the content of C, N, and O is obtained from XPS survey spectra.

**Table S3.** ORR performance comparison of the relevant Fe-based SACs

**
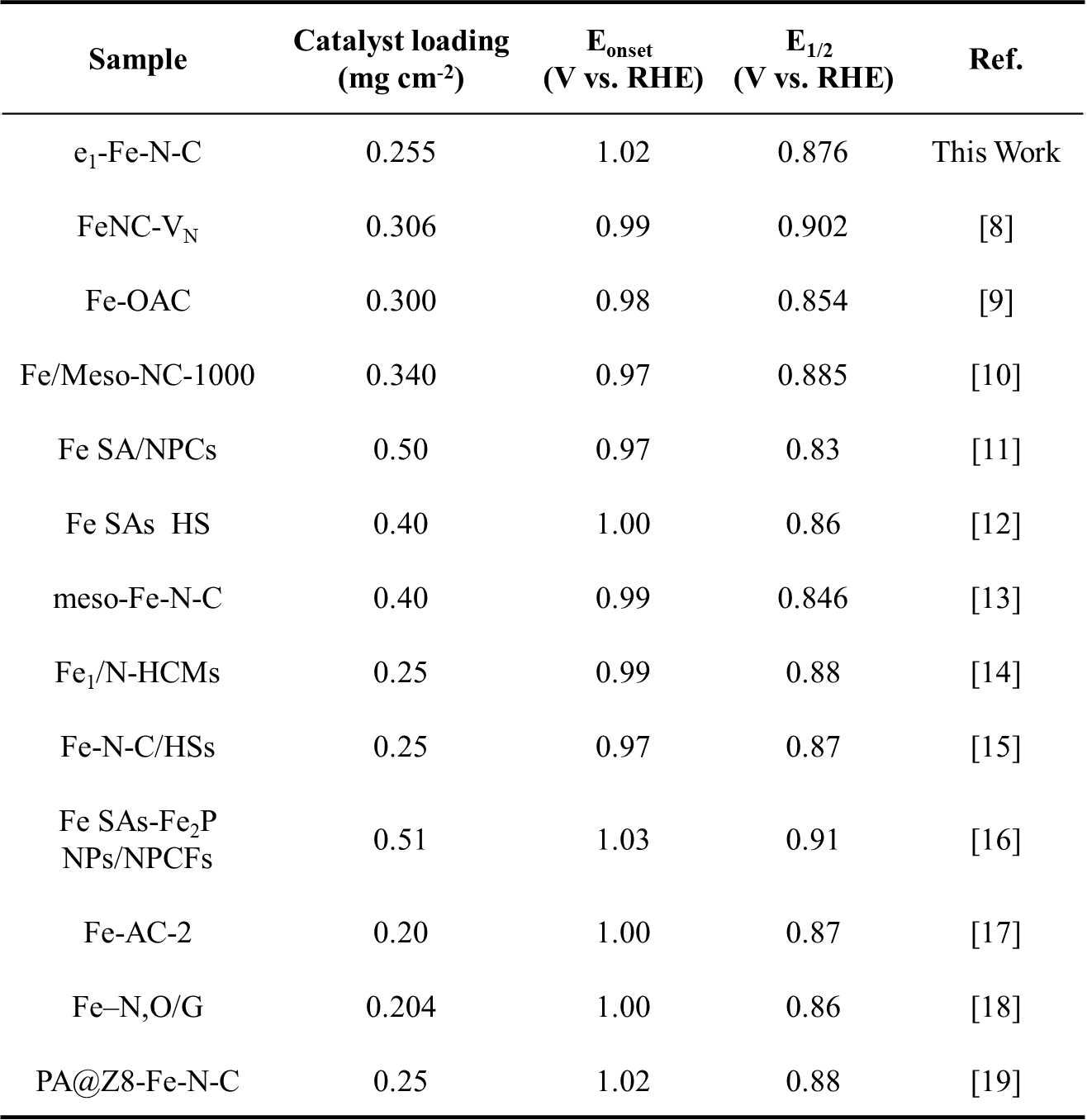
**

**Table S4.** Comparison of the ZABs properties of the e_1_-Fe-N-C catalyst with recently reported advanced catalysts.

**
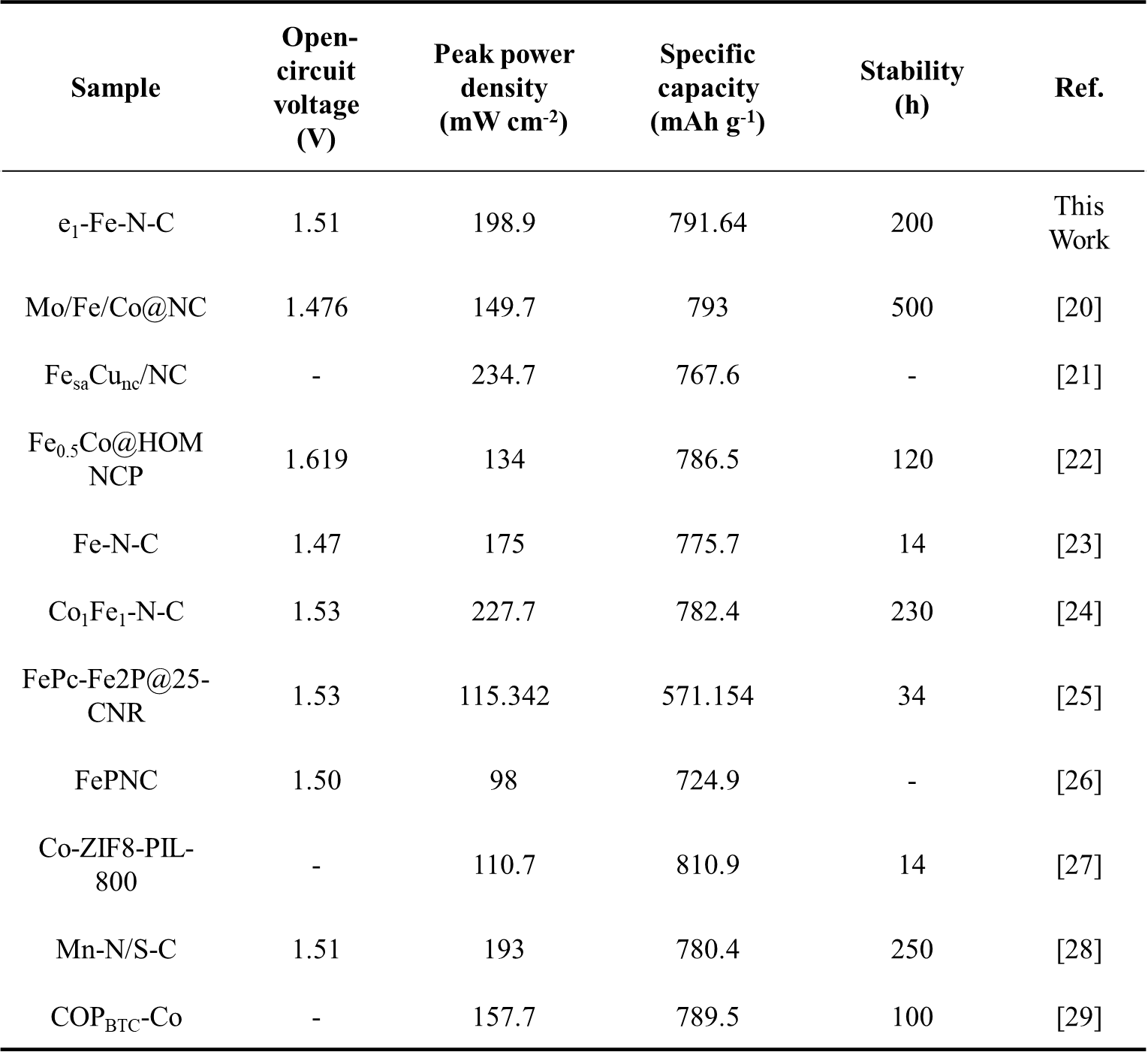
**

References

[1] G. Kresse, D. Joubert, *Phys. Rev. B* **1999**, 59, 1758.

[2] J. P. Perdew, K. Burke, M. Ernzerhof, *Phys. Rev. Lett.* **1996**, 77, 3865.

[3] S. Grimme, J. Antony, S. Ehrlich, H. Krieg, *J. Chem. Phys.* **2010**, 132, 154104.

[4] H. J. Monkhorst, J. D. Pack, *Phys. Rev. B* **1976**, 13, 5188.

[5] V. Wang, N. Xu, J.-C. Liu, G. Tang, W.-T. Geng, *Comput. Phys. Commun.* **2021**, 267, 108033.

[6] M. H. V. Huynh, T. J. Meyer, *Chem. Rev.* **2007**, 107, 5004.

[7] K. Momma, F. Izumi, *J. Appl. Crystallogr.* **2008**, 41, 653.

[8] L. Lyu, X. Hu, S. Lee, W. Fan, G. Kim, J. L. Zhang, Z. Zhou, Y. M. Kang, *J. Am. Chem. Soc.* **2024**, 146, 4803.

[9] L. B. Deng, L. Qiu, R. Hu, L. Yao, Z. J. Zheng, X. Z. Ren, Y. L. Li, C. X. He, *Appl. Catal. B-Environ.* **2022**, 305, 121058.

[10] S. N. Zhao, J. K. Li, R. Wang, J. M. Cai, S. Q. Zang, *Adv. Mater.* **2022**, 34, 2107291.

[11] H. Q. Yang, Z. Y. Li, S. Q. Kou, G. L. Lu, Z. N. Liu, *ACS Appl. Mater. Interfaces.* **2020**, 278, 119270.

[12] Y. B. Wang, P. Y. Meng, Z. H. Yang, M. Jiang, J. Yang, H. X. Li, J. Zhang, B. D. Sun, C. P. Fu, *Angew. Chem. Int. Ed.* **2023**, 62, e202304229.

[13] Y. Zhou, Y. N. Yu, D. S. Ma, A. C. Foucher, L. Xiong, J. H. Zhang, E. A. Stach, Q. Yue, Y. J. Kang, *ACS Catal.* **2021**, 11, 74.

[14] F. X. Ma, Z. Q. Liu, G. B. Zhang, H. S. Fan, Y. Du, L. Zhen, C. Y. Xu, *Small* **2023**, 19, 2207991.

[15] K. Qin, Z. Y. Zhu, F. X. Ma, J. H. Zhang, *J. Electroanal Chem.* **2022**, 906, 116021.

[16] Y. Pan, X. L. Ma, M. M. Wang, X. Yang, S. J. Liu, H. C. Chen, Z. W. Zhuang, Y. H. Zhang, W. C. Cheong, C. Zhang, X. Cao, R. A. Shen, Q. Xu, W. Zhu, Y. Q. Liu, X. D. Wang, X. J. Zhang, W. S. Yan, J. Li, H. M. Chen, C. Chen, Y. D. Li, *Adv. Mater.* **2022**, 34, 2203621.

[17] Y. P. Wang, Q. L. Li, L. C. Zhang, Y. K. Wu, H. Chen, T. H. Li, M. W. Xu, S. J. Bao, *J. Mater. Chem. A* **2021**, 9, 7137.

[18] Y. J. Li, Y. J. Ding, B. Zhang, Y. C. Huang, H. F. Qi, P. Das, L. Z. Zhang, X. Wang, Z. S. Wu, X. H. Bao, *Energy Environ. Sci.* **2023**, 16, 2629.

[19] L. S. Gao, X. Gao, P. Jiang, C. Y. Zhang, H. Guo, Y. H. Cheng, *Small* **2022**, 18, 2105892.

[20] S. L. Li, Y. T. Zhou, C. X. Xu, L. Wang, T. Z. Wang, B. K. Zhu, W. J. Xu, Y. M. A. Wu, H. C. Tao, *Small* **2024**, 2309932.

[21] C. Liang, X. Han, T. Y. Zhang, B. Dong, Y. P. Li, Z. B. Zhuang, A. J. Han, J. F. Liu, *Adv. Energy Mater.* **2024**, 2303935.

[22] W. Li, B. Liu, D. Liu, P. F. Guo, J. Liu, R. R. Wang, Y. H. Guo, X. Tu, H. G. Pan, D. L. Sun, F. Fang, R. B. Wu, *Adv. Mater.* **2022**, 34, 2109605.

[23] X. Y. Lu, H. Xu, P. X. Yang, L. H. Xiao, Y. Q. Li, J. Y. Ma, R. P. Li, L. L. Liu, A. M. Liu, V. Kondratiev, O. Levin, J. Q. Zhang, M. Z. An, *Appl. Catal. B-Environ.* **2022**, 313, 121454.

[24] H. S. Fan, X. Y. Liang, F. X. Ma, G. B. Zhang, Z. Q. Liu, L. Zhen, X. C. Zeng, C. Y. Xu, *Small* **2024**, 20, 2307863.

[25] Q. Huang, S. J. Xu, J. Liu, Y. Y. Guo, D. D. Chen, Q. H. Sun, L. J. Zhang, H. G. Nie, Z. Yang, J. J. Qian, *ACS Appl. Catal. B-Environ.* **2023**, 339, 123172.

[26] H. Liu, L. Z. Jiang, Y. Y. Sun, J. Khan, B. Feng, J. M. Xiao, H. D. Zhang, H. J. Xie, L. N. Li, S. Y. Wang, L. Han, *Adv. Energy Mater.* **2023**, 13, 2301223.

[27] J. P. Zhang, Z. Y. Mei, L. L. Yi, J. S. Tian, K. J. Li, X. C. Hu, Y. Y. Zhang, R. Wang, H. Guo, S. Q. Zang, *Appl. Catal .B-Environ.* **2023**, 338, 123044.

[28] X. Bai, Y. Wang, J. Y. Han, X. D. Niu, J. Q. Guan, *Appl. Catal. B-Environ.* **2023**, 337, 122966.

[29] C. X. Mi, H. F. Yu, L. K. Han, L. R. Zhang, L. L. Zhai, X. L. Li, Y. J. Liu, Z. H. Xiang, *Adv. Funct. Mater.* **2023**, 33, 2303235.
